# Fluoro-18-fluorodeoxyglucose positron emission tomography/computed tomography detects Ewing’s sarcoma of the larynx with multiple distant bone metastases: a case report and literature review

**DOI:** 10.3389/fmed.2023.1167350

**Published:** 2023-07-04

**Authors:** Xianwen Hu, Yan Liao, Rui Wang, Rui Wen, Dandan Li, Pan Wang, Jiong Cai

**Affiliations:** ^1^Department of Nuclear Medicine, Affiliated Hospital of Zunyi Medical University, Zunyi, China; ^2^Department of Obstetrics, Zunyi Hospital of Traditional Chinese Medicine, Zunyi, China

**Keywords:** Ewing sarcoma, larynx, computed tomography, positron emission tomography, case report

## Abstract

Ewing sarcomas (EWS) are highly malignant neoplasms of mesenchymal origin that are rare in the head and neck. Only a few laryngeal EWS have been reported in the literature. We report a 47 years-old man who visited our hospital for medical help after 5 months of hoarseness and sore throat. Computed tomography (CT) showed uneven thickening of the epiglottis fold, right vocal cord, and anterior union. In addition, fluoro-18-fluorodeoxyglucose positron emission tomography (^18^F-FDG PET)/CT has confirmed high activity in the already known laryngeal and nodal lesions, and has revealed otherwise unknown skeletal metastases. We also reviewed the published clinical features, histopathology, and imaging findings of nine patients with laryngeal EWS confirmed by pathology. The main clinical manifestations of laryngeal EWS are rapidly growing lumps, hoarseness, acute respiratory distress, and aphonia. The EWS tumor cells usually express CD99, vimentin, synaptophysin (Syn), and neuron-specific enolase (NSE) but do not express common antigen (LCA), CD20, and chromaffin granin (CgA). Laryngeal EWS’ CT imaging characteristics are mainly homogeneous, well-bounded soft-tissue masses. Our case suggests that EWS should be considered a differential diagnosis of laryngeal cancer, especially when PET/CT reveals distant bone metastasis, which is more likely to indicate EWS.

## 1. Introduction

Ewing sarcomas (EWS) are unusual, poorly differentiated, round cell tumors that occur mainly in teenagers and young people between the ages of 8 and 30 years, with a slightly greater incidence in males (1). According to the World Health Organization’s 5th classification of bone and soft-tissue tumors in 2020, EWS of bone, primitive neuroectodermal tumors, extraskeletal EWS, and Askin’s tumors are referred to collectively as the EWS family; extraskeletal EWS are rare, accounting for only 15%–20% of the EWS family (2). The most common extraskeletal EWS affect the paravertebral soft tissue, lung, stomach, kidney, and bladder; the larynx is a rare site of origin (2–5). The clinical features of laryngeal EWS are associated with tumor progression, but no clinical manifestation is obvious when the tumor is small. However, as the tumor increases in mass, it compresses the surrounding tissues or invades the recurrent laryngeal nerve, manifesting as acute respiratory distress, hoarseness, and aphasia (6). Herein, we report a case of EWS verified by pathology after a laryngeal mass was removed using an electronic laryngoscope. This case report shares our clinical diagnosis and treatment experience with EWS. Moreover, we systematically reviewed the published literature on EWS of the larynx to increase awareness of this rare disease.

## 2. Case presentation

**Figure 1:**
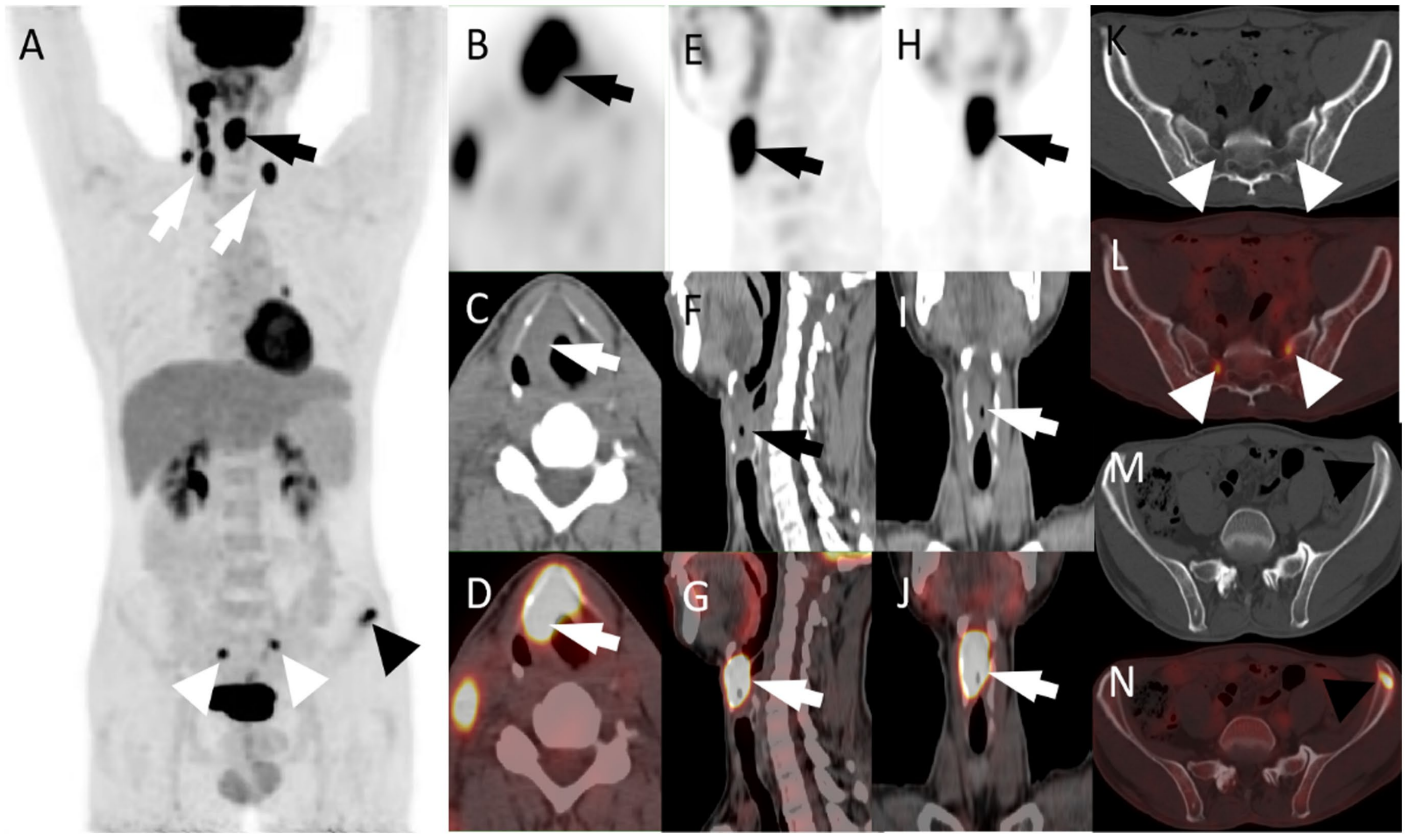
**(A)** The maximum intensity projection of the PET/CT scan shows increased ^18^F-FDG uptake in the right larynx (black arrow), bilateral supraclavicular area and right cervical area (white arrow), bilateral sacrum (white triangle), and left ilium (black triangle). (**B**, PET; **C**, CT; **D**, PET/CT fusion images) axial images of laryngeal mass. (**E**, PET; **F**, CT; **G**, PET/CT fusion images) sagittal images of the laryngeal mass. (**H**, PET; **I**, CT; **J**, PET/CT fusion images) coronal images of the laryngeal mass (SUV_max_ = 17.0; arrows). Besides, the axial CT **(K,M)** and fused PET/CT **(L,N)** images show that increased ^18^F-FDG uptake of bilateral sacrum (SUV_max_ = 8.0) and left iliac crest (SUV_max_ = 7.1) in the absence of obvious structural alterations. CT, computed tomography; ^18^F-FDG, fluoro-18-fluorodeoxyglucose; PET, positron emission tomography; SUV_max_, maximum standardized uptake value.

**Figure 2:**
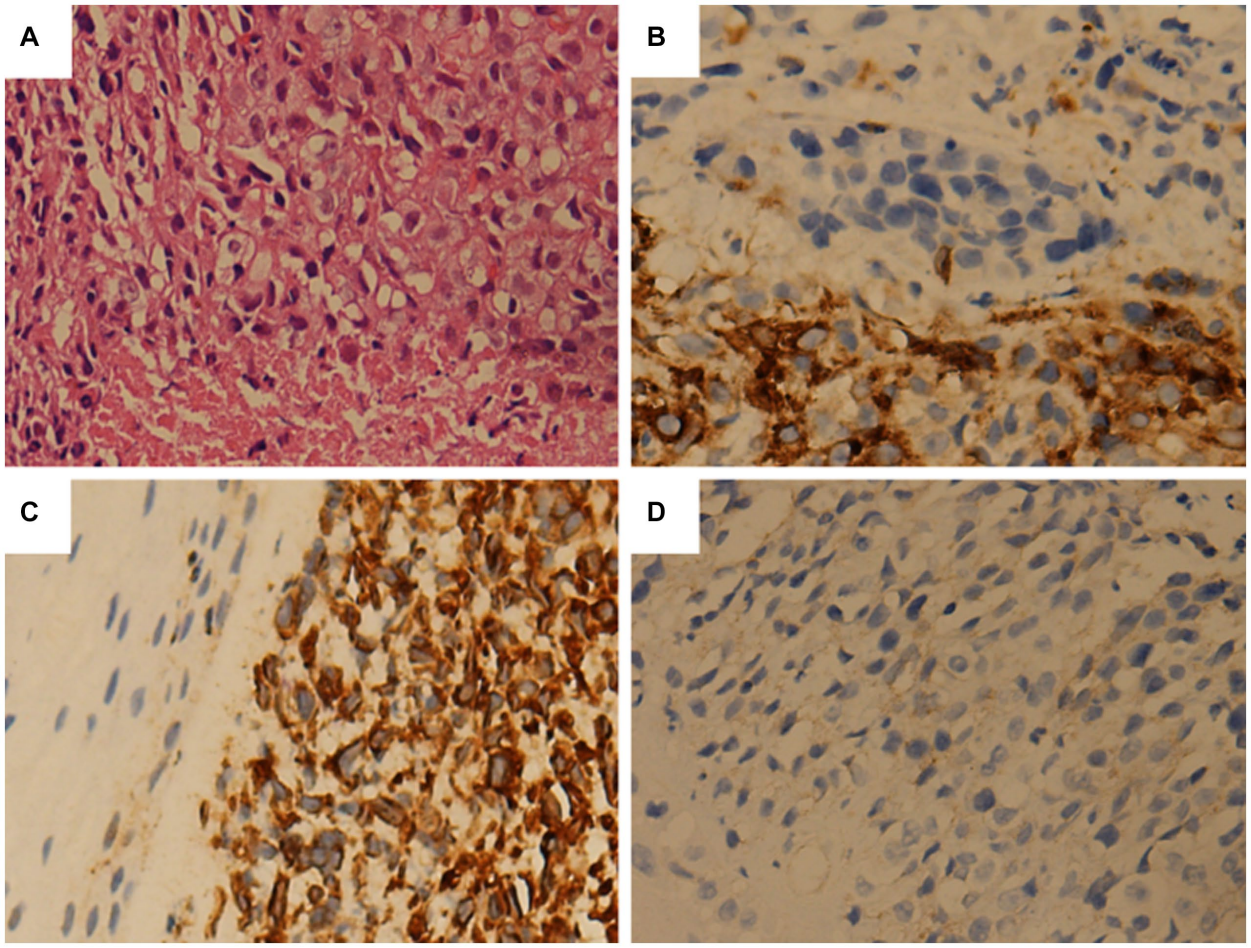
Tumor histopathology and immunohistochemical staining. **(A)** The tumor sections are stained with hematoxylin and eosin. The tumor cells are small and round, with relatively regular nuclei. **(B)** Immunohistochemistry is positive for CD99. **(C)** Immunohistochemical staining shows the positive expression of vimentin in tumor cells. **(D)** Immunohistochemistry reveals that Syn tests positive.

A 47 years-old male came to our hospital seeking medical help for a sore throat and hoarseness for 5 months without discomforts such as dyspnea or dysphagia. The patient did not receive any treatment during this period. The patient and his family had no history of tumors or other major diseases. Physical examination revealed swollen soft-tissue nodules with poor mobility on both sides of the neck. Laryngoscopy detected a homogeneous soft-tissue mass in the right glottis, involving the laryngeal chamber and the front 2/3 of the right vocal cord. A head & neck computed tomography (CT) revealed uneven thickening of the epiglottis fold, right vocal cord, and anterior union, and enlarged lymph nodes in the bilateral cervical sheath. Based on these findings, laryngeal cancer with cervical lymph node metastasis was suspected. To further assess whether the patient had distant metastases, he underwent a fluoro-18-fluorodeoxyglucose positron emission tomography (^18^F-FDG PET)/CT examination, showing increased ^18^F-FDG uptake in the corresponding lesions. Moreover, the bilateral sacrum and left iliac bone also had markedly increased radiopharmaceutical uptake (Figure 1). Based on the laryngoscopy and PET/CT findings, laryngeal cancer was suspected. The patient underwent a needle biopsy of a soft tissue mass in the larynx for accurate diagnosis and treatment. Hematoxylin-eosin staining detected small, dark, blue, round cells with transparent cytoplasm growing in patches in the tumor tissue. Immunohistochemistry (IHC) results showed that the tumor cells positively expressed synaptophysin (Syn), vimentin, CD99, and P53 and negatively expressed S100, cytokeratin (CK), and neuron-specific enolase (NSE) (Figure 2). Based on the pathology and IHC results, the patient was ultimately diagnosed with EWS. Since PET/CT showed high uptake of ^18^F-FDG in the ilium and sacrum, the patient underwent a left ilium puncture biopsy. The lesion showed the same nature as the laryngeal mass, suggesting the tumor cells had metastasized to the distal bone; therefore, the patient received a conservative treatment of vincristine, actinomycin, cyclophosphamide, etoposide, and cyclophosphamide chemotherapy. The patient was followed-up periodically during treatment. Due to deterioration, the patient died 7 months after diagnosis.

## 3. Literature review

Case reports or case series of larynx EWS published until October 31, 2022, were retrieved from the Web of Science, PubMed, and Science Direct databases, limiting the language to English. The retrieval formula was (*larynx* OR *throat*) AND (*Ewing sarcoma* OR *primitive neuroectodermal tumor*). Through detailed layer-by-layer screening, 9 patients in 9 articles met the inclusion criteria (1, 6–13). Table 1 summarizes clinical manifestations, CT and PET/CT imaging characteristics of the 10 cases of laryngeal EWS (including our patient). The main clinical manifestations of the 10 patients were rapidly growing lumps, hoarseness, acute respiratory distress, and aphonia. The CT imaging characteristics of EWS in the larynx are mainly homogeneous and well-bounded soft-tissue masses. In addition to the patient we report, only four cases reported the use of PET/CT, describing an increased ^18^F-FDG uptake in primary and/or metastatic lesions. And that, in only one out of four cases, distant metastases in nodes, lung and bone were reported. Among the cases, IHC tests showed positive expression for CD99 (7/7), vimentin (4/4), Syn (4/5), and NSE (2/3) and negative expression for leukocyte common antigen (LCA, *n* = 5), CD20 (*n* = 3), and chromogranin (CgA, *n* = 3) (Table 2).

**Table 1:** Clinical features of Ewing sarcoma of the larynx cases from the literature and our patient.

Case no. (Ref.)	First author (year, Nation)	Sex/age (years)	Size_max_ (cm)	Presentation	Imaging findings		Metastasis	IHC	Treatment	Follow-up (years)/outcome
					CT	PET				
1 (7)	Abramowsky (1983, United States)	F/0.03	1.0	Inspiratory stridor	NA	NA	No	NA	S	2/LWD
2 (8)	Jones (1995, United States)	M/0.75	NA	NA	NA	NA	No	NA	S + C	2/LWD
3 (9)	Yang (2004, Korea)	M/74	3.5	Acute respiratory distress	HSTDM	NA	No	MIC-2(+), S100(−), NSE(−), Syn(−), CgA(−), LCA(−)	S + R	0.5/LWD
4 (1)	Wygoda (2013, Poland)	M/68	2.9	Hoarseness aphonia	HoSTDM	SUV_max_ = 12.1	No	CD99(+), NSE(+), Syn(+), vimentin(+), S100(−), SMA(−), Desmin(−), LCA(−), CD56(−), CgA(−),	C + R	2.5/LWD
5 (11)	Lynch (2014, United States)	F/45	2.9	Rapidly growing lump	NA	Increased ^18^F-FDG uptake	No	CD99(+), vimentin(+), Syn(+), AE1/AE3(+), CD3(−), CD20(−), CD45(−)	C + R	NA
6 (10)	Ijichi (2016, Japan)	F/33	NA	Hoarseness	Negative	NA	No	CD99(+), NSE(+), vimentin(+), S100(+), EMA(+), Syn(+), AE1/AE3(+), LCA(−), CgA(−)	S + C	5/LWD
7 (6)	Maroun (2019, Lebanon)	M/53	5.0	Hoarseness	HSTDM	Increased ^18^F-FDG uptake	LNs, lung, bone	CD99(+)	C + R	1/LWD
8 (13)	Pasha (2020, Pakistan)	M/5	2.0	Acute respiratory	RSTDM	NA	No	CD99(+), cyclin D1(+), desmin(−), LCA(−), TdT(−), AE1/ AE3(−)	S	2/LWD
9 (12)	Wang (2022, China)	F/29	3.8	Painless mass	ISTDM	Increased ^18^F-FDG uptake	No	CD99(+), CD3(−), CD20(−), CD21(−), HMB45(−)	S + C + R	7/LWD
10	Present case	M/47	1.3	Hoarseness	HSTDM	SUV_max_ = 17.0	LNs, Bone	CD99(+), Syn(+), P53(+), vimentin(+), CD20(−), S100(−), LCA(−)	C	0.6/died

Ref, reference; CT, computed tomography; F, female; M, male; C, chemotherapy; IHC, immunohistochemistry; R, radiation therapy; S, surgery. HSTDM, heterogeneous soft-tissue density mass; HoSTDM, homogenous soft-tissue density mass; NA, not applicable; ISTDM, irregular soft-tissue density mass; SFA, strong FDG-avidity; RSTDM, rounded soft-tissue density mass; LCA, leukocyte common antigen; TdT, terminal deoxynucleotidyl transferase; NSE, neuron-specific enolase; Syn, synaptophysin; CgA, chromogranin; EMA, epithelial membrane antigen; LWD, Live without disease.

**Table 2:** Immunohistochemical staining for CD99, vimentin, Syn, NSE, LCA, CgA, and CD20 in EWS of the larynx.

Maker	Positive cases	Percent (%)
CD99	7 of 7	100
Vimentin	4 of 4	100
Syn	4 of 5	80
NSE	2 of 3	67
LCA	0 of 5	0
CD20	0 of 3	0
CgA	0 of 3	0

LCA, leukocyte common antigen; NSE, neuron-specific enolase; Syn, synaptophysin; CgA, chromogranin.

## 4. Discussion

EWS are highly aggressive malignancies. They are the second most common bone and soft-tissue sarcomas in children, more common in males than females, and peak in onset between 10 and 14 years of age (14, 15). EWS are believed to be caused by the chromosomal translocation *t*(11;22)(q24;q12), which produces an EWS-FLI1 fusion gene, disrupting transcriptional regulation, gene expression, and signal transduction (16). EWS in the larynx are rare, only nine cases have been previously reported. Laryngeal EWS lack typical clinical manifestations, presenting mainly as deep soft-tissue masses; most of the early symptoms are not obvious, but some patients may have mild local pain (12). The tumor develops rapidly and soon presents with localized swelling and severe pain, followed by localized muscle restriction and compression of the surrounding tissues. When the mass invades the surrounding recurrent and superior laryngeal nerves, symptoms including hoarseness, choking, and difficulty in breathing may result. Hematogenous metastases can occur early in most patients; the most common sites are the lung, bone, or bone marrow; local lymph node metastases can also be observed (17). Our patient was a middle-aged male, which is rare for EWS. His clinical manifestations were sore throat and hoarseness; consistent with the literature, bone and lymph node metastases were present at the time of diagnosis.

On CT, most laryngeal EWS are poorly defined, homogeneous, or heterogeneous soft-tissue density shadows, which are difficult to differentiate from other malignant tumors, such as laryngeal cancer and rhabdomyosarcoma. EWS tumor tissue has a high degree of malignancy and strong metabolism; therefore, the lesion tissue absorbs a large amount of ^18^F-FDG, showing a increased ^18^F-FDG uptake during PET/CT imaging. Compared with conventional imaging such as CT and magnetic resonance imaging PET/CT plays a better role in assessing disease extent, depicting possible occult lesions and distant metastases (18). EWS should be highly suspected when PET/CT detects distant bone metastases since they are rare in laryngeal cancer (19). Consistent with the literature, the CT findings of our patient involved the epiglottic fold, right vocal cord, a soft-tissue mass shadow with uneven thickening of the anterior commissure, and an unclear boundary with the adjacent normal tissue. In addition, PET/CT has confirmed high activity in the already known laryngeal and nodal lesions, and has revealed otherwise unknown skeletal metastases. Notably, only 1 of the 9 published cases of laryngeal EWS was diagnosed with distant metastasis at diagnosis, which is inconsistent with the high heterogeneity of EWS. Of these reported laryngeal EWS, four patients described their PET/CT findings, all showing a high ^18^F-FDG uptake, with one describing its specific SUV_max_ value of 12.1, which is consistent with the ^18^F-FDG PET/CT findings of our patient. The inclusion of a whole-body examination like PET/CT in the initial disease evaluation, may avoid the risk of understaging by missing distant metastases, as demonstrated in our patient who exhibited sacrum and ilium localizations. Therefore, PET/CT may represent a complementary imaging modality and may provide additional information on disease extent and occult sites of metastases, leading to disease upstage and with potential impact on treatment management.

An accurate diagnosis of EWS depends on histopathological examination. Microscopically, the tumor cells appear as small, round, blue cells with clear cytoplasm and vacuolation. IHC can distinguish EWS from other small cell tumors, including neuroblastoma, rhabdomyosarcoma, lymphoma, small cell carcinoma, and other microscopically small round cell tumors. More than 90% of EWS positively express surface antigen MIC2 (CD99), a 32-kD membrane glycoprotein involved in cell adhesion, migration, and apoptosis. However, this marker is not specific; it is also detected in poorly differentiated synovial sarcoma and lymphoblastic lymphoma. EWS can be excluded by negative staining for LCA, desmin, MyoD1, smooth muscle actin, CK, and epithelial membrane antigen (1). Moreover, neuroblastoma can be excluded by negative staining for CD56, S100 proteins, synaptophysin, and pheochromogranin, as well as by the absence of rosette formation and neurofibrillary matrix (1, 2). Based on our patient and published data, CD99, vimentin, and Syn are expressed positively in most patients with EWS of the larynx; LCA, CgA, and CD20 are not expressed. Therefore, these tumor markers are helpful for the differential diagnosis of primary laryngeal EWS.

EWS of the larynx are known to be sensitive to radiotherapy; however, owing to their rarity, no consensus on their clinical management has been reached. Like most malignancies, the treatment of laryngeal EWS is dominated by surgical excision, with radiotherapy and chemotherapy performed as auxiliary empirical therapies (20). Previous studies have shown that patients who undergo radiotherapy after surgery have better prognoses; furthermore, a smaller diameter of the largest tumor correlates with a greater survival rate (12, 21, 22). Moreover, the prognoses of laryngeal EWS are related to the patient’s age and disease stage at the time of diagnosis. Patients with metastasis at diagnosis and older age have poor prognoses (23). Overall, the prognosis of extraosseous EWS is better than that of bone EWS; the 5 years survival rate can reach 60% (24). Reviewing the published literature, nine cases of laryngeal EWS had good prognoses, and no tumor progression or recurrence was found during the follow-up period; however, most of the patients had a short follow-up period. Therefore, further research must be conducted with longer-term follow-ups and larger sample sizes. Our patient had developed metastases of the lymph nodes and distant bone at the time of diagnosis; he died due to the deterioration of his condition in the late stage of chemotherapy.

## 5. Conclusion

This rare case involved an adult male patient with laryngeal EWS and multiple distant bone metastases. PET/CT can play a substantial role in diagnosing and managing laryngeal EWS. Especially when distant bone metastasis is found, the diagnosis of EWS should be considered. Unfortunately, Laryngeal EWS has a poor prognosis when diagnosed with distant bone metastases; therefore, further exploration of effective treatment options is mandated.

## Data availability statement

The original contributions presented in the study are included in the article/supplementary material, further inquiries can be directed to the corresponding authors.

## Ethics statement

Written informed consent was obtained from the individual(s) for the publication of any potentially identifiable images or data included in this article.

## Author contributions

JC: funding acquisition. DL and RWang: investigation. XH and RWen: methodology. XH and YL: writing-original draft. XH, PW, and JC: writing-review and editing. All authors contributed to the article and approved the submitted version.

## Funding

This study was funded by the National Natural Science Foundation of the People’s Republic of China, NSFC (grant number: 82260353).

## Conflict of interest

The authors declare that the research was conducted in the absence of any commercial or financial relationships that could be construed as a potential conflict of interest.

## Publisher’s note

All claims expressed in this article are solely those of the authors and do not necessarily represent those of their affiliated organizations, or those of the publisher, the editors and the reviewers. Any product that may be evaluated in this article, or claim that may be made by its manufacturer, is not guaranteed or endorsed by the publisher.
